# Different patterns of amygdala priming differentially affect dentate gyrus plasticity and corticosterone, but not CA1 plasticity

**DOI:** 10.3389/fncir.2013.00080

**Published:** 2013-05-03

**Authors:** Rose-Marie Vouimba, Gal Richter-Levin

**Affiliations:** ^1^CNRS, Unité Mixte de Recherche 5287, Institut de Neurosciences Cognitives et Intégratives d'Aquitaine, Université de BordeauxTalence, France; ^2^Department of Psychology, University of HaifaMount Carmel, Haifa, Israel; ^3^The Sagol Department of Neurobiology, University of HaifaMount Carmel, Haifa, Israel; ^4^Institute for the Study of Affective Neuroscience, University of HaifaMount Carmel, Haifa, Israel

**Keywords:** hippocampus, dentate gyrus, BLA, LTP, corticosterone, stress

## Abstract

Stress-induced activation of the amygdala is involved in the modulation of memory processes in the hippocampus. However, stress effects on amygdala and memory remain complex. The activation of the basolateral amygdala (BLA) was found to modulate plasticity in other brain areas, including the hippocampus. We previously demonstrated a differential effect of BLA priming on long-term potentiation (LTP) in the CA1 and the dentate gyrus (DG). While BLA priming suppressed LTP in CA1, it was found to enhance it in the DG. However, since the amygdala itself is amenable to experience-induced plasticity it is thus conceivable that when activity within the amygdala is modified this will have impact on the way the amygdala modulates activity and plasticity in other brain areas. In the current study, we examined the effects of different patterns of BLA activation on the modulation of LTP in the DG and CA1, as well as on serum corticosterone (CORT). In CA1, BLA-priming impaired LTP induction as was reported before. In contrast, in the DG, varying BLA stimulation intensity and frequency resulted in differential effects on LTP, ranging from no effect to strong impairment or enhancement. Varying BLA stimulation patterns resulted in also differential alterations in Serum CORT, leading to higher CORT levels being positively correlated with LTP magnitude in DG but not in CA1. The results support the notion of a differential role for the DG in aspects of memory, and add to this view the possibility that DG-associated aspects of memory will be enhanced under more emotional or stressful conditions. It is interesting to think of BLA patterns of activation and the differential levels of circulating CORT as two arms of the emotional and stress response that attempt to synchronize brain activity to best meet the challenge. It is foreseeable to think of abnormal such synchronization under extreme conditions, which would lead to the development of maladaptive behavior.

## Introduction

Stress-induced alterations in learning and memory processes are involved in the pathophysiology of stress-related disorders. Yet, stress effects on learning and memory are not only deleterious, but range from impairment to no effect to facilitation. The various effects of stress on memory functions may depend on stressors characteristics and on stress-induced release of stress hormones and neuromodulators (Shors, 2001; Baldi and Bucherelli, 2005; Joëls and Baram, 2009). Electrophysiological approaches revealed that long-term potentiation (LTP), a synaptic model of memory (Bliss and Collingridge, 1993), is also differentially altered by various stressors (Foy et al., 1987; Mesches et al., 1999; Kavushansky et al., 2006). The complexity of stress effects on hippocampal-dependent memory processes emerges also from its differential impact on hippocampal sub-regions. Thus, while stress and stress hormones have consistently been reported to impair LTP in CA1 (Pavlides et al., 1996; Xu et al., 1998; Vouimba et al., 2006), the outcome on dentate gyrus (DG) LTP is more diversified (Bramham et al., 1998; Gerges et al., 2001; Yamada et al., 2003). For instance, discriminatory avoidance learning, which increases arousal, selectively inhibited LTP in CA1 but enhanced LTP in DG (Izaki and Arita, 1996). Predator stress enhanced LTP in DG for 5–7 days (Dringenberg et al., 2008), but impaired CA1 LTP (Vouimba et al., 2006). Moreover, increased release of CORT impaired CA1 LTP (Diamond et al., 1992; Pavlides et al., 1993), but had no effect on LTP in DG (Bramham et al., 1998).

The amygdala, an essential component of the neural network involved in emotional memory and stress-related behaviors, is a key mediator of stress effects on hippocampal-dependent memory processes (Roozendaal et al., 2003, 2004). Thus, while lesions or pharmacological suppression of the basolateral amygdala nucleus (BLA) were reported to impair DG LTP (Abe 2001), Kim et al. (2001) reported normal CA1 LTP in BLA lesioned stressed animals. Similarly, while BLA stimulation augments LTP in DG (Akirav and Richter-Levin, 1999), we later reported that BLA priming impairs CA1 LTP (Vouimba and Richter-Levin, 2005; Vouimba et al., 2007), further validating the claim that amygdalar modulation of hippocampal plasticity differs among its sub-fields.

Thus, an intact and active amygdala seems to be necessary for stress-induced alteration of hippocampal processes (Chauveau et al., 2008). However, although stress has been demonstrated to readily activate the amygdala, its effects on amygdala activity are rather complex. For instance, an acute platform stress enhanced BLA activity and LTP (up to 7 days), while repeated stress exposure inhibited BLA LTP (Vouimba et al., 2004). Thus, different stressors could activate the amygdala in ways that could be accountable for the differential effects of stress on memory processes (Tsoory et al., 2008). Furthermore, the amygdala itself is amenable to experience-induced plasticity. It is thus conceivable that when activity within the amygdala is modified this will have impact on the way the amygdala modulates activity and plasticity in other brain areas.

In the present study, we further addressed the role of the BLA on CA1 and DG LTP by testing the impact of various patterns of BLA priming on hippocampal plasticity. In addition, since it has been indicated that amygdala activation may lead to the activation of the hypothalamic-pituitary-adrenal (HPA) axis (Goldstein et al., 1996; Feldman and Weidenfeld, 1998) we have also measures serum corticosterone (CORT) following the different patterns of BLA activation. We report that various patterns of BLA activation mainly inhibited LTP in CA1 but differentially modulated LTP in DG. In addition, various patterns of BLA activation enhanced CORT to different levels such that CORT levels positively correlated with LTP magnitude in DG but not in CA1.

## Materials and methods

### Subjects

Experiments were performed on male Sprague–Dawley rats (200–250 g), housed in Plexiglas cages and maintained on a free-feeding regimen with a 12:12 h light/dark schedule. Experiments were performed during the light phase of the cycle at least 1 week after their arrival from the supplier (Harlan Laboratories, Jerusalem, Israel). The experiments were performed in accordance with University of Haifa Ethics and Regulations and complied with the National Institute of Health guide for Care and Use of laboratory animals (NIH publication number 8023).

### Surgery

Rats were anesthetized with Equitisin (42.5 mg/ml chloral hydrate, 21 mg/ml magnesium sulfate, and 9.7 mg/ml pentobarbital sodium; 0.4 ml/100 g ip) and mounted onto a stereotaxic frame (Stoelting, Wood Dale, IL, USA) and the head position was adjusted to place bregma and lambda in the same horizontal plane. The scalp was incised and retracted and small burr holes were drilled in the skull for the placement of stimulating and recording electrodes. Body temperature was monitored and maintained at 37°C ± 0.5 by a regulated heating pad during the course of the experiments.

### Evoked field potentials in CA1 and DG

A recording glass electrode (tip diameter, 2–5 μm; filled with 2 M NaCl) was stereotaxically positioned in the CA1 [4.2 mm posterior to bregma (AP), 2.5 mm from midline (ML), and ~2 mm dorsoventral (DV) or in the DG (4.2 mm AP, 2.5 mm ML, and ~3.7 mm DV)]. Bipolar concentric stimulating electrodes (125 μm; Kopf, Tujunga, CA, USA) were inserted in the ipsilateral BLA (3 mm AP, 5.2 mm ML, and 7.6 mm DV) and either in the controlateral ventral hippocampal commissure (vHC: 2 mm AP, 1.5 mm ML, and ~3 mm DV) for activating field potentials in the CA1 or in the ipsilateral perforant pathway (PP: 8 mm AP, 4 mm ML, and ~3 mm DV) for activating field potentials in DG. The DV location of the recording and stimulating electrodes was adjusted to maximize the amplitude of evoked field potentials (EPs). A reference electrode consisting of a 100 μm coated wire was affixed to the skull in the area overlapping the nasal sinus.

### Stimulating and recording procedures

CA1 and DG field potentials evoked by single pulses delivered to vHC or PP, respectively (0.1 ms rectangular monophasic pulses) were amplified (×1000) by a A-M systems amplifier, displayed on an oscilloscope, digitized at 10 kHz (CED) and stored to disk for off-line analysis (Signal-2 software). Baseline responses were established by means of stimulation intensity (50–200 μA) sufficient to elicit a response representing ~30% of the maximal amplitude of the EPs.

LTP was assessed by measuring the increase in the population spike amplitude (PS) of the EPs for both DG and CA1.

### Protocols

Baseline recording of EPs in CA1 or DG was conducted for 30 min (one pulse every 30 s). In the *Control* group baseline recording in CA1/DG was followed by application of a theta-like high frequency stimulation (TS): one set of 5 trains; each train consisted of 5 pulses at 100 Hz; inter-train interval was 200 ms to the vHC or PP for LTP induction in CA1 or DG, respectively. After TS, responses to test pulse stimuli were recorded every 20 s for an additional 1 h.

In the *BLA priming* groups, baseline recording was followed by various patterns of BLA stimulation 30 s prior TS. Responses to low frequency baseline pulses to the vHC or PP were then collected every 30 s for 1 h. We used 8 experimental groups which differed by BLA stimulation frequency or strength.

### Patterns of amygdala stimulation

Stimulation *strength* was modulated by varying the number of pulses in the protocol. Four Strength protocols were used: the first protocol consisted of 2 trains of five pulses at 100 Hz; intertrain interval, 200 ms; 1 V, 50 μs pulse duration (*BLA 2 trains*). The second, third, and fourth protocols “*BLA 10 trains*,” “*BLA 15 trains*,” and “*BLA 20 trains*,” respectively, consisted of constant frequency and pulse duration throughout the protocols, with a gradual increase of the number of trains to 10, 15, and 20, respectively.

BLA Stimulation *frequency* was modulated by varying train frequencies. Five protocols were used. Two high frequency stimulation (HFS) protocols: “*BLA 100 Hz*” consisting of 10 trains of five pulses at 100 Hz; intertrain interval, 200 ms; 1 V, 50 μs pulse duration (identical to “*BLA 10 trains*”). “*BLA 400 Hz*” was identical to the previous group except that the pulses were delivered at 400 Hz. *Three low frequency stimulation (LFS) protocols:* “*BLA 25 Hz*” and “*BLA 1 Hz*” consisted of 10 trains of five pulses (50 pulses) at 25 and 1 Hz, respectively. In addition, we used a classic LFS protocol (900 pulses at 1 Hz: “*BLA 1 Hz, 900*”) known to readily elicit long-term depression as well as depotentiation in several brain regions (Wang et al., 1997; Vouimba et al., 2000).

### Serum corticosterone

To assess the effect of BLA stimulations on serum CORT and establish a possible relation between CORT and hippocampal plasticity; in another sub-set of animals, stimulating and recording electrodes were positioned in the BLA for stimulation and in vHC-CA1 or PP-DG for LTP induction, as described above. In control animals, the stimulating electrode was lowered in the amygdala but no stimulation was then administered. Change in synaptic plasticity in all groups was recorded for 10 min, then 5 min later (15 min following amygdala activation in experimental groups), animals were decapitated, trunk blood collected into plastic tubes, and allowed to clot for 1 h before centrifugation (3000 rpm for 15 min at 4°C). Serum was aliquoted into storage microfuge tubes (Eppendorf) and stored at −80°C until assayed for CORT levels, using enzyme immunosorbent assay kit (Assay Designs, Ann Arbor, MI, USA) according to manufacturer's instructions. The sensitivity for the CORT assay was ~1.3 ng/ml, and the average inter- and intra-assay covariance (%) was less than 10 and 5%, respectively. CORT results are presented as ng/mL. All blood samples were obtained during the light cycle (between 8:30 and 10:30 a.m.) at the nadir of the diurnal CORT rhythm.

### Histology

After completion of the studies, animals were either transcardially perfused with physiological saline, followed by 10% buffered formalin (electrophysiological study) or brains were directly remove after blood collection (CORT study). Brains were then post-fixed in formalin-saccharose 30% solution for at least 3 days and subsequently frozen, cut coronally on a sliding microtome into 80 μm sections. The sections were mounted on gelatin-coated slides, and stained with cresyl violet for microscopic examination of the placements of the electrode in the BLA.

### Data analysis

The amplitude of the PS of the EPs was expressed as the mean percentage (±SEM) of the individual basal values of animals for each group. Groups differences were analyzed by ANOVA and *post-hoc* Fisher tests. A simple regression analysis was used to determine correlation between LTP magnitude and CORT. All statistical analyses were carried out with Statview.

## Results

### Histology

**Figure 1:**
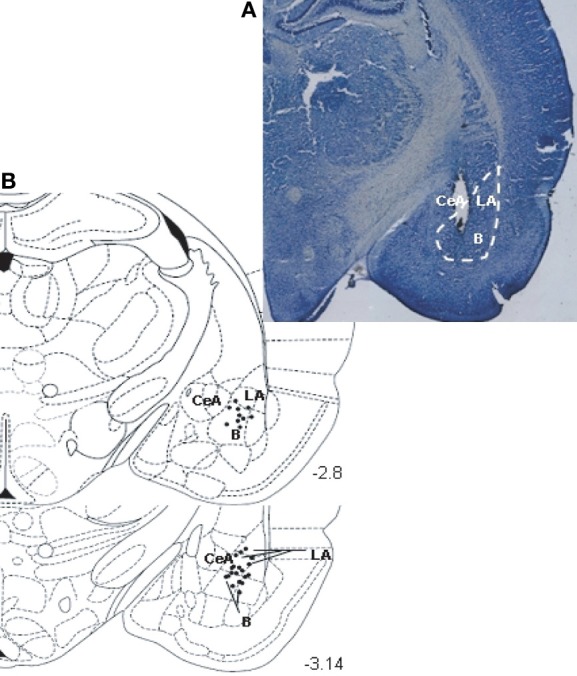
**(A)** Photomicrograph illustrating the positioning of a stimulating electrode tip in BLA. Brain section at about 3.14 mm posterior to bregma. **(B)** A diagram depicting a coronal section of the rat brain (at position 3.14 mm posterior to bregma) showing the location of the stimulating electrode tip in the BLA, for an average pool of 30 animals (LA, lateral amygdala; B, basal amygdala; CeA, central amygdala). Diagram is from the stereotaxic atlas of Paxinos and Watson (1997).

Histological analysis showed that the stimulating electrode was mainly located in the BLA (Figure 1). Only rats with correct electrode positioning in the BLA were included for further analysis. The indicated numbers of rats per groups are the final group sizes after histological control of electrode location.

### Effects of frequency modulation of BLA activation on LTP in CA1 and DG

**Figure 2:**
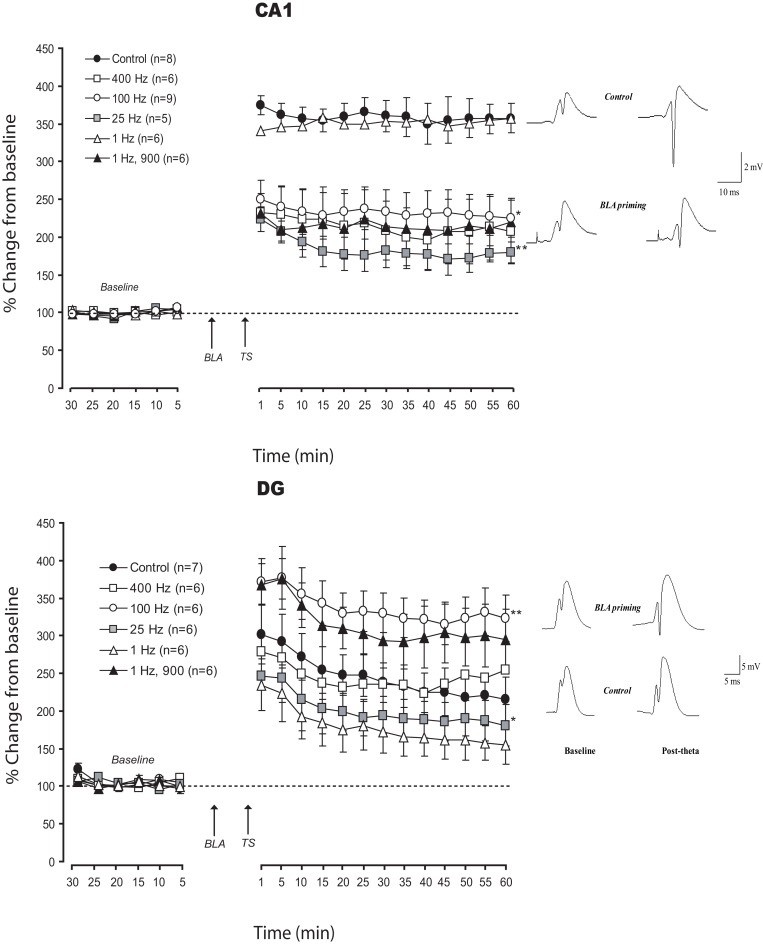
**Effects of frequency modulation of BLA activation on LTP in CA1 and DG: Mean (±SEM) percentage of baseline.** The majority of patterns of BLA activation used here impaired LTP in CA1. In DG, the different patterns induced changes varying from enhancement to strong impairment. The pattern less favorable for DG (1 Hz) was the only one which did not interfere with CA1 plasticity. Only one pattern (25 Hz) induced similar deficit in DG and CA1 LTP. *Post-hoc* Fischer test: ^*^p < 0.05 and ^**^p < 0.001 significantly different from control groups.

In the attempt to understand the effects of frequency modulation of amygdala activation on LTP in CA1 and DG, we used low and high frequency BLA stimulation patterns (LFS and HFS- Figure 2). Priming the BLA with HFS, 100 or 400 Hz, impaired LTP in vHC-CA1 pathway (% changes for the last 10 min: *CONT*: 357.1 ± 21.6%; *BLA-100 Hz:* 227.4 ± 26.6%; *BLA-400 Hz:* 209.5 ± 35.4%). ANOVAs performed on these data revealed a significant difference between control and BLA priming groups [*F*_(2, 20)_ = 8.1; *p* < 0.01]. No significant difference was observed between the *BLA-100 Hz* and *BLA-400 Hz* groups (*p* = 0.45).

When BLA was primed using LFS, 25 Hz and classic LFS pattern (900 pulses at 1 Hz) impaired LTP in the CA1, while 1 Hz (50 pulses) had no effect on LTP (% changes for the last 10 min: *BLA-25 Hz:* 178.6 ± 14.5%; *BLA-1 Hz:* 353.8 ± 16.36%). ANOVAs revealed a significant difference between groups [*F*_(3, 21)_ = 19.7; *p* < 0.0001]. *Post-hoc* Fisher tests showed a significant difference between *BLA-25 Hz* and both *control* and *BLA-1 Hz* (*p* < 0.0001), as well as between *BLA-1 Hz, 900* and both *control* and *BLA-1 Hz* groups (*p* < 0.0001) without any difference between *control* and *BLA-1 Hz* (*p* = 0.65).

As previously reported (Vouimba and Richter-Levin, 2005; Vouimba et al., 2007) priming the BLA with 100 Hz HFS enhanced LTP in DG (% changes for the last 10 min: *CONT*: 213.3 ± 27.9%; *BLA-100 Hz:* 320 ± 30%). However, a 400 Hz HFS did not affect LTP (% changes for the last 10 min: *BLA-400 Hz:* 243.2 ± 36.2%). ANOVA revealed a significant difference between groups [*F*_(2, 16)_ = 3.9; *p* < 0.05]. A *post-hoc* Fisher test revealed a significant difference between 100 Hz and both CONT and 400 Hz (*p* < 0.0001) with no significant difference between CONT and 400 Hz (*p* = 0.95).

When the BLA was primed with LFS, both 1 and 25 Hz patterns impaired LTP in the DG compared to control (% changes for the last 10 min: *BLA-25 Hz:* 181.2 ± 27.3%; *BLA-1 Hz:* 153.3 ± 24.5%). In addition, unlike in the vHC-CA1 pathway, 900 pulses at 1 Hz enhanced LTP in DG (% changes for the last 10 min for *BLA-1 Hz,900*: 298 ± 33%). Statistical analysis revealed a significant difference between groups [*F*_(3, 20)_ = 4.5; *p* = 0.014]. Pairwise comparisons showed a significant LTP reduction in both *BLA-25 Hz* and *BLA-1 Hz* as compared with *control* (*p* < 0.01). LTP in the *BLA-1 Hz, 900* pulses was significantly enhanced as compared to the *control* group (*p* < 0.001).

### Modulation of strength of BLA activation on LTP in CA1 and DG

**Figure 3:**
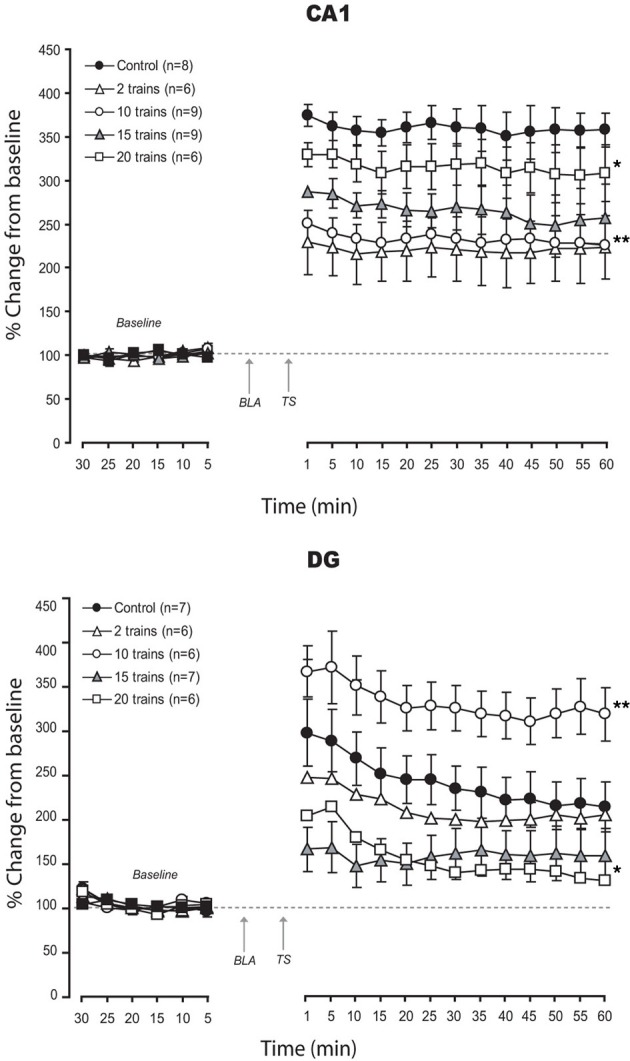
**Effects of strength modulation of BLA activation on LTP in CA1 and DG: Mean (±SEM) percentage of baseline.** Modulation BLA activation strength induced linear impairment in CA1 LTP, with weakest activation associated with stronger deficit. In contrast, in DG, non-linear effects, rangind from enhancement to strong impairment of LTP were found. *Post-hoc* Fischer test: ^*^p < 0.05 and ^**^p < 0.001 significantly different from control groups.

Results are seen on Figure 3.

Varying the strength of BLA activation, we found consistent impairment of vHC-CA1 LTP with all patterns used. Interestingly, the strongest impairment was seen with the weakest pattern (*BLA-2 trains*) while lighter changes accompanied stronger patterns of stimulation (% changes for the last 10 min: *CONT*: 357.1 ± 21.6%; *BLA-2 trains:* 222.3 ± 37%; *BLA-10 trains:* 227.4 ± 26.6%; *BLA-15 trains:* 252.8 ± 39%; *BLA-20 trains:* 306.86 ± 32%). ANOVA revealed a significant difference between groups [*F*_(4, 33)_ =4.97; *p* = 0.003]. A *post-hoc* Fisher test showed a significant difference between control and all BLA priming groups (*p* < 0.05). Significant pairwise comparisons revealed significant differences between the BLA priming groups (*p* < 0.05) except for 2 trains and 10 trains which were not significantly different from each other (*p* = 0.7).

Unlike the CA1, changes in LTP in the PP-DG pathway were non-linear (% changes for the 10 last min: *CONT*: 213.3 ± 27.9%; *BLA-2 trains:* 201 ± 22.6%; *BLA-10 trains:* 320 ± 30%; *BLA-15 trains:* 156.5 ± 31%; *BLA-20 trains:* 132 ± 10.4%). ANOVA revealed a significant difference between groups [*F*_(4, 27)_ = 7.85; *p* < 0.001]. A *post-hoc* Fisher test showed a significant difference between control and all BLA priming groups (*p* < 0.05), except for the 2 trains group, which was not significantly different from control (*p* = 0.06). Significant pairwise comparisons revealed significant differences between BLA priming groups (*p* < 0.05) except for the 15 trains vs. 20 trains groups, which were not significantly different from each other (*p* = 0.8).

In summary, comparing the effects of different patterns of BLA stimulation we found a dichotomy between CA1 and DG. The less efficient pattern for CA1 was best for DG and vice versa [except for two stimulation parameters (25 Hz and 15 trains) which impaired LTP in both CA1 and DG].

### Amygdala stimulation increased levels of serum corticosterone

Amygdala stimulation is known to enhance serum CORT (Dunn and Orr, 1984; Vouimba et al., 2007). Here we examined if different patterns of BLA activation could differentially influence CORT. We selected BLA stimulation parameters which yield the strongest effects on both CA1 and DG plasticity. Thus, we investigated the effects of 400, 100, 25, 1 Hz, and 1 Hz,900 on CORT.

**Figure 4:**
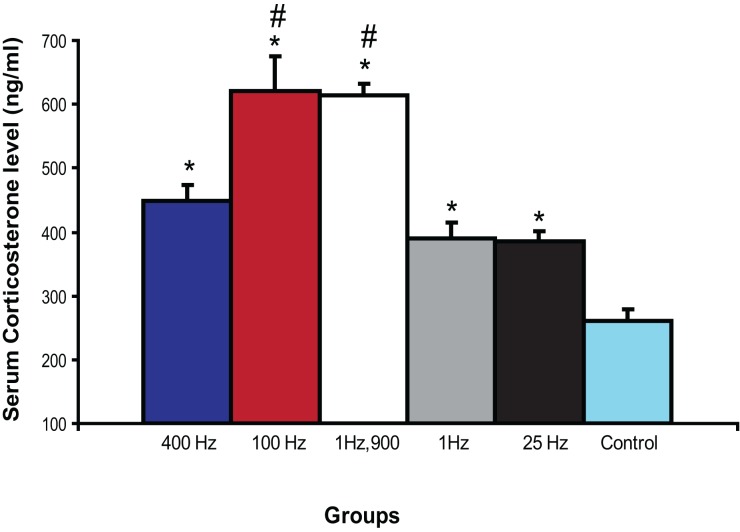
**Effect of BLA activation on serum corticosterone.** BLA-stimulated rats showed significantly increased levels of CORT compared with control rats (^*^p < 0.01). In addition, 100 Hz and 1 Hz 900 groups showed significantly more CORT compared with 1, 25, and 400 Hz (^#^*p* < 0.001).

Serum samples analyses revealed a significant difference among groups [*F*_(5, 28)_ = 24.35; *P* < 0.0001; Figure 4]. The five BLA stimulation protocols used in this study significantly increased CORT as compared with control rats (*p* < 0.01). In addition, a *post-hoc* Fisher test showed that both 100 Hz and 1 Hz,900 groups exhibited significantly higher CORT level than the other stimulated groups (*p* < 0.001).

### Correlation between serum corticosterone and hippocampal LTP

Regression analyses revealed that the amplitude of DG LTP was positively correlated with CORT in BLA-stimulated animals (*r* = 0.75; *p* < 0.0001). In contrast, no linear relation was observed between CA1 LTP and CORT (*r* = 0.004; *p* = 0.98).

## Discussion

Consistent with our previous findings (Vouimba and Richter-Levin, 2005; Vouimba et al., 2007), BLA priming revealed a functional dichotomy between effects on LTP in vHC-CA1 synapses and LTP in the PP-DG pathway. Modulation of the strength and frequency of BLA priming stimulation induced changes in LTP in both CA1 and DG. However, while in CA1 BLA priming inhibited LTP, in the DG the effects ranged from impairment to no effect to enhancement of plasticity. Such changes are reminiscent of the effects of various stressors on LTP in the hippocampus.

In accordance with that, modulation of the strength and frequency of BLA priming stimulation resulted in different levels of serum CORT such that CORT levels positively correlated with LTP in DG but not in CA1. Our findings provide further evidence for a differential amygdalar control of plasticity in hippocampal sub-regions. They also provide insight into how various stressors through differential activation of the amygdala may alter memory processes in brain circuitry required for tasks execution.

It is interesting to note that variability in the outcome of stress and of BLA activation is reflected in the DG and not in the CA1. Traditionally, with respect to learning and memory, focus was more on the CA3–CA1 area of the hippocampus, partly because of the impact of the relatively selective ischemic lesions to CA1 (Yamashima et al., 2007) and due to the quality of place cells in these regions (Rolls, 2010). The current results are consistent with recent proposals for a differential role of the DG in aspects of memory, such as pattern separation (Gilbert et al., 2001; Kesner, 2007; Aimone et al., 2011; Schmidt et al., 2012). They add to this view the possibility that DG-associated aspects of memory will be enhanced under more emotional or stressful conditions (Kogan and Richter-Levin, 2008; Segal et al., 2010).

The BLA is known to play a key role in many facets of emotional processing (Fanselow and LeDoux, 1999). Exposure to emotionally arousing experiences enhances BLA neuronal activity. For instance, foot-shock was reported to enhance and synchronize the firing rate of BLA neurons (Pelletier et al., 2005). Moreover, fear-provoking experiences induced lasting potentiation in the BLA (McKernan and Shinnick-Gallagher, 1997; Rogan et al., 1997; Garcia et al., 1998; Vouimba et al., 2004). Correspondingly, learning under stress was found to activate the MAP kinase signal transduction cascade within the BLA (Akirav et al., 2001; Kogan and Richter-Levin, 2008). In humans, neuro-imaging studies and electrophysiological recordings demonstrated strong and reproducible activation of the amygdala in response to fearful stimuli (Whalen et al., 1998; Morris et al., 2002; Oya et al., 2002).

However, stress does not universally enhance BLA neuronal activity. Varying the intensity of an acute stress can lead to different levels of activation of various markers of activity and plasticity in the BLA; e.g., ERKII, CREB (Kogan and Richter-Levin, 2008; Ilin and Richter-Levin, 2009). Some stressors may decrease or have no effect on the BLA. For instance, both acute tail-shocks stress and re-exposure to the stressful context was reported to suppress spontaneous unit activity in the BLA (Shors, 1999). Further, a single exposure or re-exposure to a moderate platform stress inhibited long-lasting LTP in the BLA (Vouimba et al., 2004; Kavushansky et al., 2006). Importantly, clinical studies have found that the amygdala response to fearful faces habituated rapidly over repeated presentations of aversive stimuli (Breiter et al., 1996; Büchel et al., 1998; LaBar et al., 1998).

Stress-induced modulation of BLA activity has been involved in stress modulation of memory processes. Indeed, manipulations which reduce or enhance BLA excitability, respectively, impair or facilitate memory storage (Dickinson-Anson and McGaugh, 1993; Packard et al., 1994; Hatfield and McGaugh, 1999) and synaptic plasticity in the hippocampus (Ikegaya et al., 1996; Akirav and Richter-Levin, 1999; Kim et al., 2001, 2005; Vouimba and Richter-Levin, 2005; Vouimba et al., 2007).

Electrical stimulation of the amygdala is known to produce, in rodents, a complex pattern of behavioral and autonomic changes that resemble fear or aggressive behavior. For example, a brief 200 Hz stimulation of the amygdala increases the aggressiveness of male Syrian golden hamsters. The effect was sensitive to stimulation amplitude and frequency and lasted for about 30 min with a peak 10–15 min after stimulation (Potegal et al., 1996). The authors suggested that the persistence of aggression could result from LTP-like changes within the amygdala-related neural circuitry. In humans, amygdala stimulation also elicits feelings of fear, anxiety, and terror (Chapman et al., 1954; Gloor et al., 1981). Early study from Mark and Ervin (1970) showed for example that stimulation of the amygdala with different intensity (≤5 mA) induced either defensive or aggressive behavior. More recently electrical stimulations (50 Hz, pulse duration 1 ms, 5 s duration; Intensity 0–2.5 mA) of the right amygdala, in humans, were reported to induce negative emotions, especially fear and sadness. In contrast, stimulations of the left amygdala were able to induce either pleasant (happiness) or unpleasant (fear, anxiety, sadness) emotions (Lanteaume et al., 2007). It is conceivable that by changing the stimulation characteristics, the authors could have induced a different set of emotions. Although we cannot link our different protocols to a specific behavior, together with the above mentioned studies, our data suggest that the different amygdala priming protocol may be related to different emotional state. Thus, stressors-induced different activation patterns at the BLA can be accountable for the wide variety of stress effects on hippocampal LTP and memory processes. Both the nature of the stressor and its temporal attributes could result in differential activation of the amygdala and in a different outcome with regards to changes in both DG and CA1.

Anatomically, there are strong direct and indirect reciprocal connections between the BLA and the hippocampus. However, within the dorsal hippocampal circuitries, the amygdala seems to modulate information processing polysynaptically via its projections to the entorhinal cortex, parasubiculum, and subiculum (Pikkarainen et al., 1999; Pitkänen et al., 2000). Though the different hippocampal sub-regions has been proposed to work together to build a unified memory (Squire and Zola-Morgan, 1991), activation of the BLA, by means of its efferent connections, may favor the selection of some circuits in detriment of others and lead to a qualitatively and quantitatively different memory (Richter-Levin, 2004; Vouimba and Richter-Levin, 2005; Segal et al., 2010).

**Figure 5:**
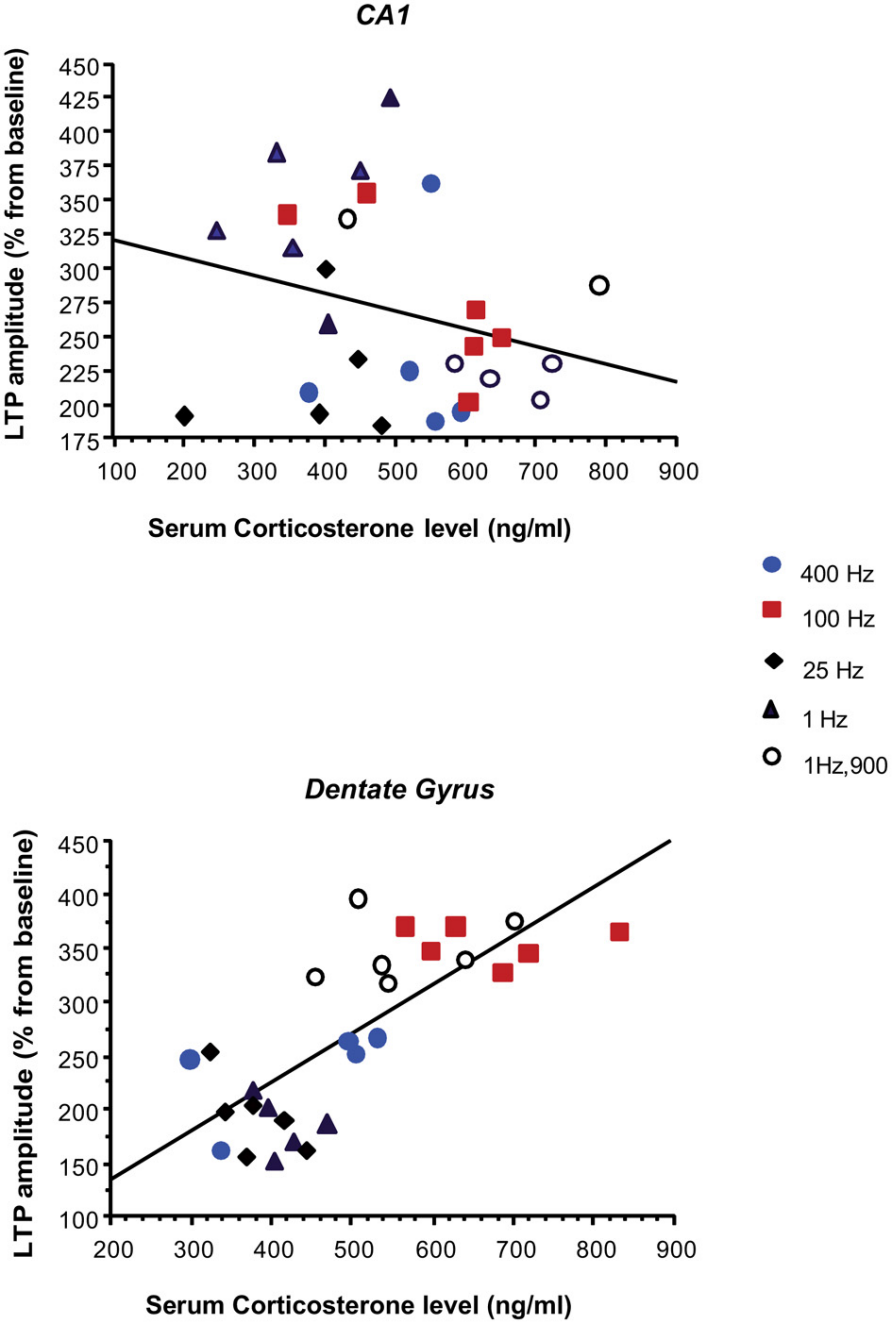
**Relationship between the magnitude of LTP in the PP-DG and vHC-CA1 pathways and serum corticosterone levels in BLA-stimulated rats.** Amplitude of the PP-DG LTP was positively correlated with CORT levels (*r* = 0.76; *P* < 0.0001), while no significant correlation was observed between LTP in vHC-CA1 pathway and CORT level (*r* = 0.27; *p* = 0.16). Control animals were not included in the correlation.

Another interesting finding was that BLA activation increased CORT levels and that higher levels of CORT were obtained with patterns which also enhanced LTP in DG but not in CA1 (Figure 5). In the control group, CORT level was not correlated with the magnitude of LTP in DG suggesting that amygdala activation is a prerequisite. The amygdala seems to dynamically modulate both CORT and DG-LTP highlighting a sensitivity of both parameters to stressors characteristics, as opposed to the lack of sensitivity and monotonous mode of response of CA1. This result complements our previous findings disclosing dissociation in the mechanism by which the BLA influences hippocampal LTP in DG and CA1. We showed that BLA modulation of LTP in DG, but not in CA1, was dependent on glucocorticoid transmission (GC) in the BLA. Indeed, blocking BLA GC receptors impaired amygdala-dependent enhancement of DG-LTP but had no effect on the impairing effect of BLA on CA1 LTP (Vouimba et al., 2007). Dissociated and bi-directional effects of CORT on DG and CA1 LTP were also reported in studies in which CORT was directly applied in an *in vitro* hippocampal preparation (Pu et al., 2007).

The complexity of effects of stress on learning and memory is also reflected in the effects of CORT on these processes (de Quervain et al., 2009). CORT, released upon exposure to a stressful event, is known to either enhance or impede memory performance (Sandi et al., 1995; Akirav et al., 2004; de Quervain et al., 2009; Chauveau et al., 2010) as well as activity and plasticity in the hippocampus (McEwen, 1994; Pavlides and McEwen, 1999; Alfarez et al., 2002) and the amygdala (Duvarci and Paré, 2007; Mitra and Sapolsky, 2008). Interestingly, the ability of GC to alter memory processes was found to depend on the cooperation of stress hormones, noradrenaline (NA), and CORT within the BLA (McGaugh, 2000; McGaugh and Roozendaal, 2002). Indeed, while GC and NA agonists into the BLA-facilitated memory formation in a variety of learning paradigms (Roozendaal and McGaugh, 1997) subsequent intra-BLA infusions of their antagonists blocked the memory-enhancing effects (Quirarte et al., 1997; Roozendaal and McGaugh, 1997; Roozendaal et al., 2002). Studies suggest that GC exerts a time-dependent permissive action on the efficacy of the NA system to promote memory (Roozendaal, 2000; Roozendaal et al., 2002). Thus, it is possible that BLA-induced increase in CORT, which alters activity and plasticity in BLA, CA1, and DG, may be mediated in part, via interaction with NA.

However, as was suggested before, the outcome of emotional, stressful, or traumatic experiences would depend on even broader range of interactions (Joëls and Baram, 2009).

In this study, we showed that weak and strong protocols have a differential impact on both DG and CA1 LTP. These protocols have been labeled strong or weak based on the frequency and number of pulses in the protocols. However, the outcome of these protocols, in term of BLA activation is difficult to appraise since we did not measure their direct impact on BLA neuronal activity. Studies from Paré et al. (2003) showed that the amygdala is endowed with a strong inhibitory interface (intercalated cells masses -ITC-) which gates impulse traffic between its input and output stations. Stimulation of the BLA was shown to induce, through differential activation of the ITC, both enhancement and inhibition of the main amygdala output. It is therefore not excluded that what we considered a weak pattern may in fact activate whether than inhibit the BLA, depending of their outcome on the ITC. This is congruent with the high level of CORT induced by the 1 Hz, 900 protocol.

It is interesting to think of BLA pattern of activation and the differential levels of circulating CORT as two arms of the emotional and stress response that attempt to synchronize brain activity to best meet the challenge (Kogan and Richter-Levin, 2008; Segal et al., 2010). It is foreseeable to think of abnormal such synchronization under extreme conditions, which would lead to the development of maladaptive behavior.

### Conflict of interest statement

The authors declare that the research was conducted in the absence of any commercial or financial relationships that could be construed as a potential conflict of interest.
